# Left ventricular untwist determines intradialytic hemodynamics and outcomes in mildly reduced and preserved ejection fraction patients

**DOI:** 10.14814/phy2.70609

**Published:** 2025-11-02

**Authors:** Nidhal Bouchahda, Fabian Scheipl, Wissal Rouetbi, Kouloud Hafi, Mohamed Yessine Kallela, Aymen Najjar, Nouha Ben Mahmoud, Meriam Ben Salem, Maissa Hadj Ibrahim, Sallemi Habib, Hajer Mani, Sabra Aloui, Mejdi Ben Messaoud, Habib Skhiri

**Affiliations:** ^1^ Cardiology A Department, Research Laboratory LR12 SP 16 Fattouma Bourguiba University Hospital, Monastir University Monastir Tunisia; ^2^ Munich Center for Machine Learning Ludwig‐Maximilians‐Universitat Munchen Munich Germany; ^3^ Nephrology Department Fattouma Bourguiba University Hospital, Monastir University Monastir Tunisia; ^4^ Nephrology Department Tahar Sfar University Hospital, Monastir University Monastir Tunisia; ^5^ El Frina Hemodialysis Center Monastir University Monastir Tunisia; ^6^ MDS Hemodialysis Center Monastir University Monastir Tunisia

**Keywords:** chronic kidney diseases, functional data analysis, hemodialysis, hypotension, longitudinal strain, torsion

## Abstract

Hypotension during hemodialysis (HD) is common in patients with reduced ejection fraction (EF), but its occurrence in those with mildly reduced or preserved EF is less clear. We hypothesized that intravascular hypovolemia during HD impairs hemodynamics when left ventricular (LV) untwisting is compromised. We studied 70 patients on maintenance HD ≥2 years (mean age 50 ± 15; 41% female), all with LVEF ≥40%. Echocardiography assessed global longitudinal strain (GLS) and LV torsion, analyzed with functional data methods. Lower systolic blood pressure (SBP) was independently associated with older age (*p* = 0.001) and higher ultrafiltration (*p* = 0.009), while larger inferior vena cava diameter correlated with higher SBP (*p* = 0.007). Greater diastolic untwist was significantly associated with higher SBP (global *p* = 0.034), whereas GLS showed no significant association (global *p* = 0.098). During a median 9.2‐month follow‐up, six patients (8%) died. LV torsion was associated with lower mortality (global *p* = 0.10), with significant effects across the diastolic untwist phase. In contrast, more negative GLS during diastole was associated with increased mortality (global *p* = 0.03). These findings suggest that better diastolic untwisting may improve hemodynamic stability and outcomes in HD patients with preserved or mildly reduced LVEF.

## Graphical abstract

In patients undergoing hemodialysis with mildly reduced or preserved left ventricular ejection fraction, stronger diastolic LV untwisting supports blood pressure maintenance and is associated with improved survival. Impaired untwist, reflecting delayed diastolic recoil, predisposes to intradialytic hypotension and adverse outcomes. These findings identify LV untwist as a key physiological determinant of hemodynamic tolerance to fluid removal during dialysis.

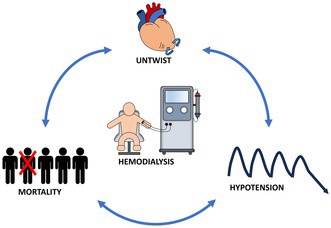

## 1 INTRODUCTION

Intradialytic hypotension is a serious complication associated with increased mortality and poor‐quality dialysis (Flythe et al., 2015). Its pathophysiology is complex, involving a decline in blood volume (BV), impaired vascular resistance, and reduced cardiovascular reserve (Sars et al., 2020). While intradialytic hypotension is expected in patients with reduced ejection fraction, its occurrence in those with preserved left ventricle (LV) ejection fraction is less understood. During hemodialysis, when fluid withdrawal exceeds the rate of refilling from the interstitium, intravascular hypovolemia occurs. This leads to a significant decline in venous return, which in turn reduces cardiac output and blood pressure (Sars et al., 2020). To maintain an adequate cardiac output during hypovolemia, improved LV diastolic function is crucial. Timely relaxation of longitudinal fibers and untwisting of helices create a diastolic depression generating low intra‐LV pressure during diastole (Badano & Muraru, 2019). This low pressure ensures a suction effect that maintains effective LV filling despite hypovolemia. Once the LV is properly filled, a sufficient stroke volume is achieved, ensuring good organ perfusion. The aim of this study is to investigate how LV mechanics modulate hemodynamics during maintenance hemodialysis in patients with preserved LV ejection fraction.

## 2 MATERIALS AND METHODS

Patients undergoing maintenance hemodialysis were recruited in four Tunisian centers. Inclusion criteria were defined as (1) Dialysis vintage ≥2 years; this duration was considered necessary to ensure only stable patients were included. (2) Sinus rhythm patients with LV ejection fraction ≥40%. Non‐inclusion criteria were defined as (1) severe valvular heart diseases, (2) unstable coronary artery diseases, and (3) infectious states.

The study obtained the approbation of the ethics committee of the Faculty of Medicine of Monastir and all patients gave informed written consent.

### 2.1 Hemodialysis session

Each patient underwent a comprehensive transthoracic echocardiographic exam at baseline and then was followed during the next three hemodialysis sessions. During each session, a total of seven systolic blood pressure (SBP) measurements were recorded as follows: before embranchment, right after embranchment, four times during hemodialysis, and once at the end of the session.

The parameters for conducting hemodialysis, including the ultrafiltrate rate and duration, were left to the discretion of the patient and their medical staff.

### 2.2 Strain imaging

Transthoracic echocardiographic measurements were acquired on a VIVID E9 (GE Healthcare, Waukesha, WI, USA) device using an M5 probe and stored on an offline workstation. Strain analysis was performed on EchoPAC V113 software. A frame rate >50 frames per second was used to obtain all strain measurements.

Standard longitudinal strain analysis was acquired on four, three, and two chamber views of the LV (Figure S1).

Torsion strain was acquired from basal and apical levels. The basal view was obtained at the level of the mitral valve from a parasternal short axis view. To acquire the apical view, the probe was positioned at a 4‐chamber view that tilted down to have a circular apical cut of the LV with either none or the slightest view of the right ventricle. Care was taken to have circular‐shaped views. Regions of interest were manually adjusted to guarantee optimal tracking. Echogenic pericardium was excluded. After applying circular strain, rotational values were extracted. LV twist was calculated as the difference between apical and basal rotational values throughout the cardiac cycle (Badano & Muraru, 2019; Sabatino et al., 2021) (Figure S2).

### 2.3 Strain values

We used the Store Trace option from ECHOPAC software, which allowed the curves depicting LV longitudinal strain and LV torsion to be exported as numerical series. Next, the data were normalized by setting the traces to equal length (33 sample length), correcting for variation in heart rate and number of frames per cycle.

### 2.4 Outcome

The primary outcome was SBP measurements during hemodialysis. The secondary outcome was all‐cause mortality within 25 months after baseline assessment.

### 2.5 Statistics

Categorical data were expressed as absolute numbers and percentages.

Continuous variables were summarized as mean ± SD for normally distributed data, and as median with interquartile range (IQ) for non‐normally distributed data.

To include the strain curve as a covariate, functional data analysis was employed (Crainiceanu et al., 2024). A multilevel scalar‐on‐function regression was used to predict SBP (Goldsmith et al., 2011; Scheipl et al., 2015). Functional covariates included LV torsion curve, GLS curve, and right ventricular free wall strain curve. Scalar covariates comprised age, sex, diabetes, hypertension, left ventricular mass indexed, dialysis vintage, ultrafiltrate volume, as well as patient as a random effect. Confidence intervals for the functional estimates were constructed after verifying assumptions and further reinforced using bootstrap methods. A significance level of *p* < 0.05 was adopted. All‐cause mortality was also modeled using scalar‐on‐function regression with a binomial family and logit link function. Due to the limited number of events, the primary analysis was conducted without adjustment for scalar covariates to minimize the risk of overfitting. Only the global longitudinal strain and left ventricular torsion curves were included as predictors. A sensitivity analysis adjusting for age was performed and is presented in the Supplementary Material. In scalar‐on‐function logistic regression, coefficient functions β(t) represent log‐odds. For interpretability, example odds ratios (ORs) were calculated and presented in the Results section. The analysis was conducted using R software with the refund, mgcv, and tf packages used to implement the results (Goldsmith et al., 2010; Wood, 2017).

## 3 RESULTS

We included a total of 70 patients with an average age of 50.4 ± 14.9 years, of which 29 (41%) were female (Table 1).

**TABLE 1:** Patients' characteristics.

	Patient (*n* = 70)
Age, years, mean (SD)	50.4 (14.9)
Sex, female, *n* (%)	29 (41.4%)
Hypertension, *n* (%)	46 (65.7%)
Diabetes, *n* (%)	7 (10.0%)
Dialysis vintage, years, median (IQ)	4 (6–10)
Weight, kg, mean (SD)	69.9 (13.2)
Ultrafiltrate, mL, mean (SD)	2350 (663)
LVEF, %, mean (SD)	60 (6)
Inferior vena cava diameter, mm, mean (SD)	16.3 (3.15)
TAPSE, mm, mean (SD)	23.9 (3.64)
Left ventricular mass indexed, g/m^2^, mean (SD)	111 (39.0)
*E*/*E*', mean (SD)	10.6 (4.62)
LV torsion max, degree, mean (SD)	25.5 (8.4)
Time to LV torsion max, %, median (IQ)	0.453 (0.05)
GLS max, %, mean (SD)	−20.0 (3.83)
Time to GLS max, %, mean (SD)	0.463 (0.05)
Follow up, months, median (IQ)	9.2 (7.6–24.6)
Death, *n* (%)	6 (8)

Abbreviations: *E*/*E*', early diastolic transmitral flow velocity/early diastolic mitral annular velocity; GLS, global longitudinal strain; IQ, interquartile interval; LV, left ventricle.

**FIGURE 1:**
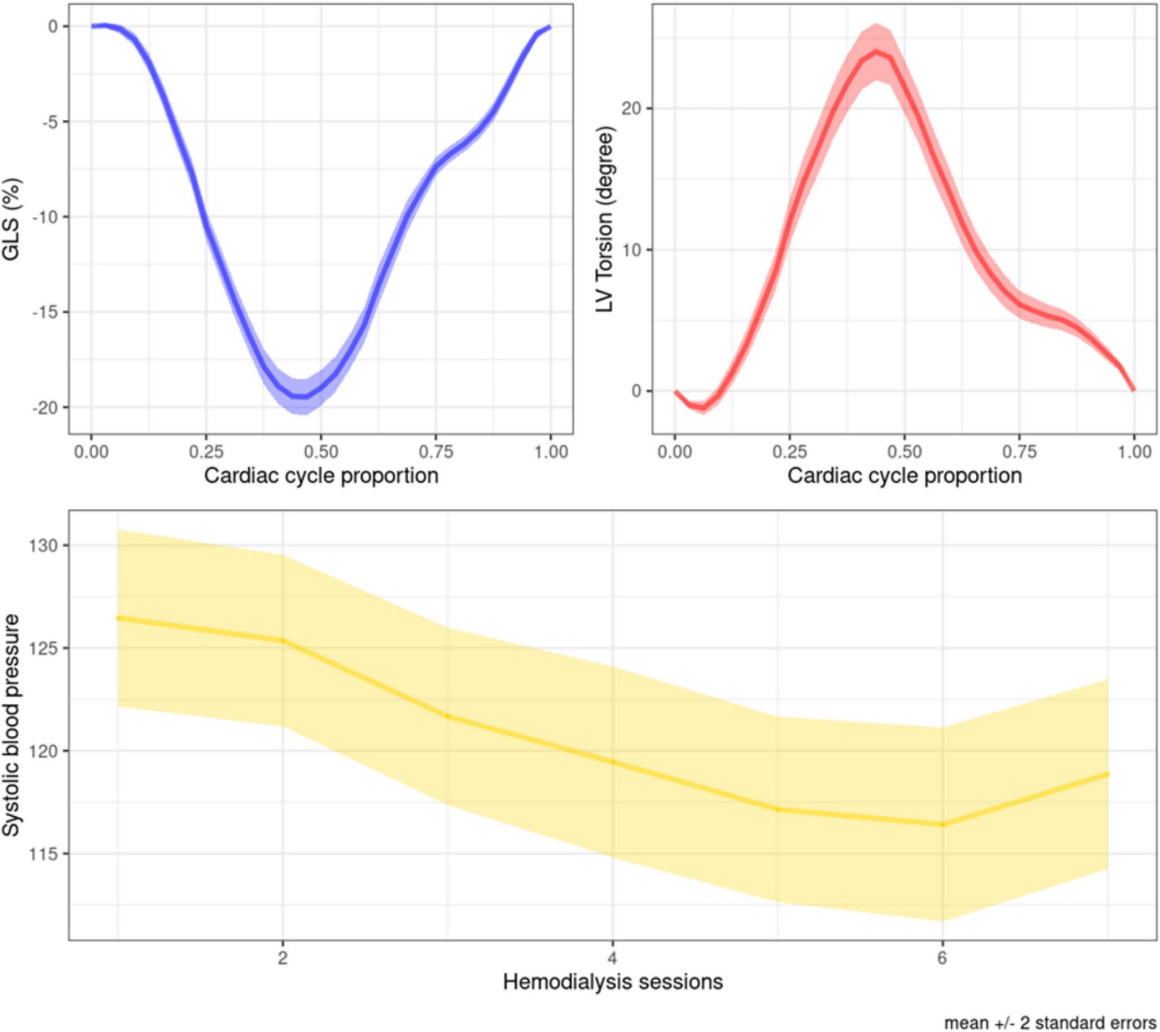
Mean curves for the global longitudinal strain (upper left), the left ventricular torsion (upper right), and systolic blood pressure during dialysis (bottom). GLS, global longitudinal strain. Light ribbons show (pointwise) ±2 standard errors.

Hypertension and diabetes were present in 46 (65.7%) and 7 (10%) patients, respectively. The average dialysis vintage was 7.84 (5.90) years. Three patients had a mildly reduced ejection fraction while the remaining had a preserved ejection fraction. The mean ultrafiltrate volume was 2350 (663) mL. The indexed LV mass averaged 111 (39.0) g/m^2^. Peak LV torsion was of 25.5 (8.40) degree occurring at 0.45 (0.05) proportion of the cardiac cycle. Negative peak GLS was −20.0 (3.83) % occurring at 0.46 (0.05) proportion of the cardiac cycle (Table 1). Figure 1 illustrates the mean GLS and LV torsion curves.

### 3.1 Predicting SBP during hemodialysis

Mean SBP during the three hemodialysis sessions at each of the seven measurement times is illustrated in Figure 1. As expected, there was a systematic tendency to decrease.

The scalar on function regression to predict SBP indicated that, among classical predictors included in the model, age and ultrafiltrate volume were negatively and significantly associated with SBP, while IVC diameter was significantly and positively associated with SBP. The remaining covariates were not significant as illustrated in Table 2.

**TABLE 2:** Predictors of systolic blood pressure during hemodialysis.

	Coefficient	95% CI		*p*
Ultrafiltrate, mL	−0.001	−0.003	−0.0005	0.004
Sex	1.4	−7.8	10.6	0.82
Age, years	−0.30	−0.60	−0.006	0.04
Hypertension	5.85	−2.73	14.4	0.18
Diabetes	0.97	−0.93	2.8	0.9
Inferior vena cava diameter, mm	1.33	0.09	2.57	0.03
Dialysis vintage, years	−0.45	−1.27	0.37	0.28
Left ventricular mass indexed, g/m^2^	0.12	−0.02	0.26	0.09

**FIGURE 2:**
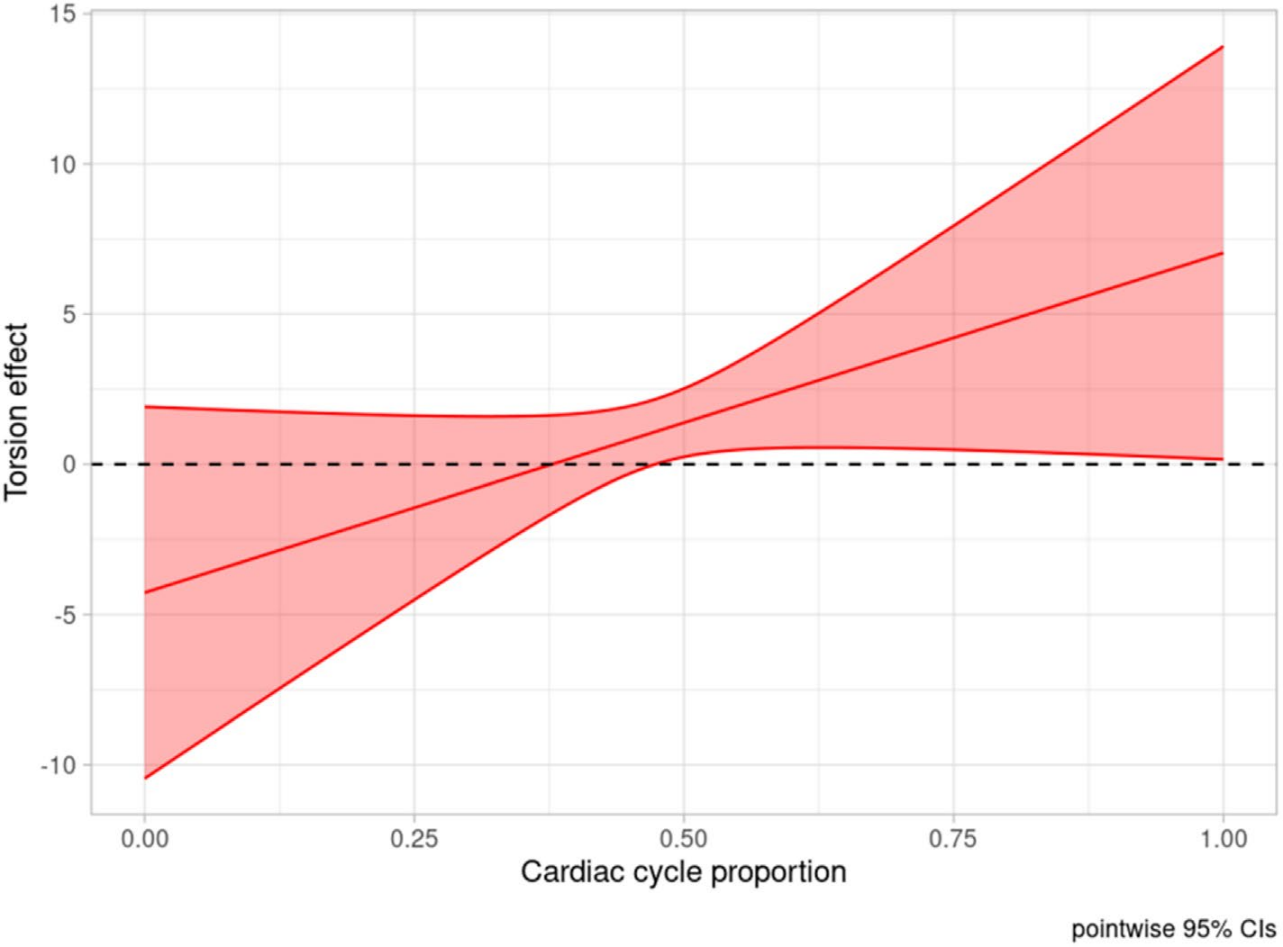
Left ventricular torsion effect on systolic blood pressure during dialysis with (pointwise) confidence interval. The horizontal dashed line corresponds to 0. Effect is significant at the cardiac cycle proportion where the confidence interval does not contain 0.

**FIGURE 3:**
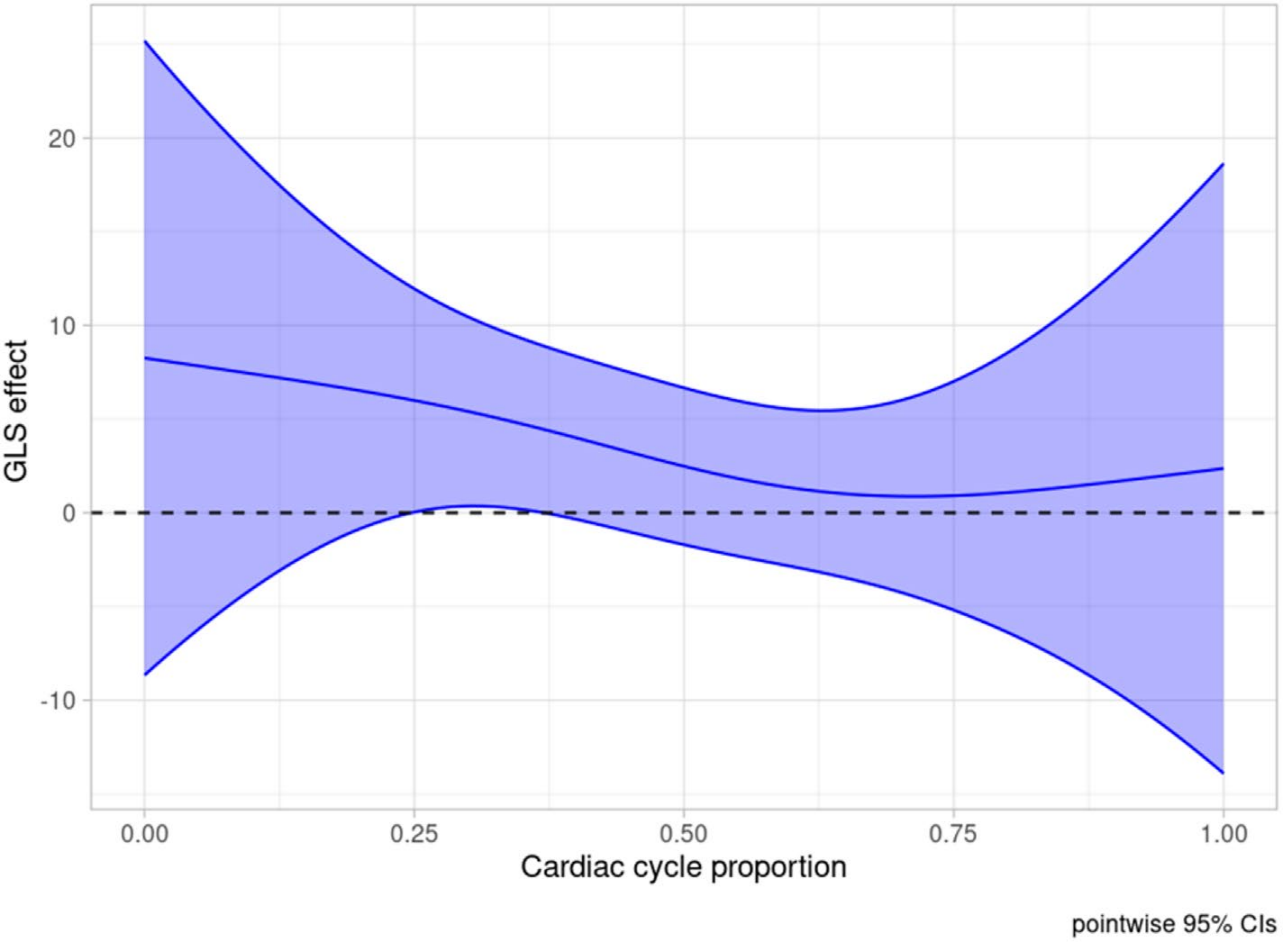
Global longitudinal strain effect on systolic blood pressure during dialysis with its confidence interval. The horizontal dashed line corresponds to 0. Effect is significant at the cardiac cycle proportion where the confidence interval does not contain 0. GLS, global longitudinal strain.

Regarding the functional predictors, diastolic untwist during the second half of the cardiac cycle was positively and significantly associated with SBP (Figure 2, global *p* value: 0.034), while GLS failed to show a (globally) significant association with SBP (Figure 3, global p‐value: 0.098).

The overall variance explained by the model was 70%.

### 3.2 Predicting all‐cause mortality

Patients were followed up for a median time of 9.2 months (IQR: 7.6–24.6), up to a maximum of 25 months. Death occurred in 6 patients (8%). To reduce model complexity for this small number of events, only GLS and LV torsion were used to predict all‐cause 24‐month mortality without adjusting for other patient characteristics.

**FIGURE 4:**
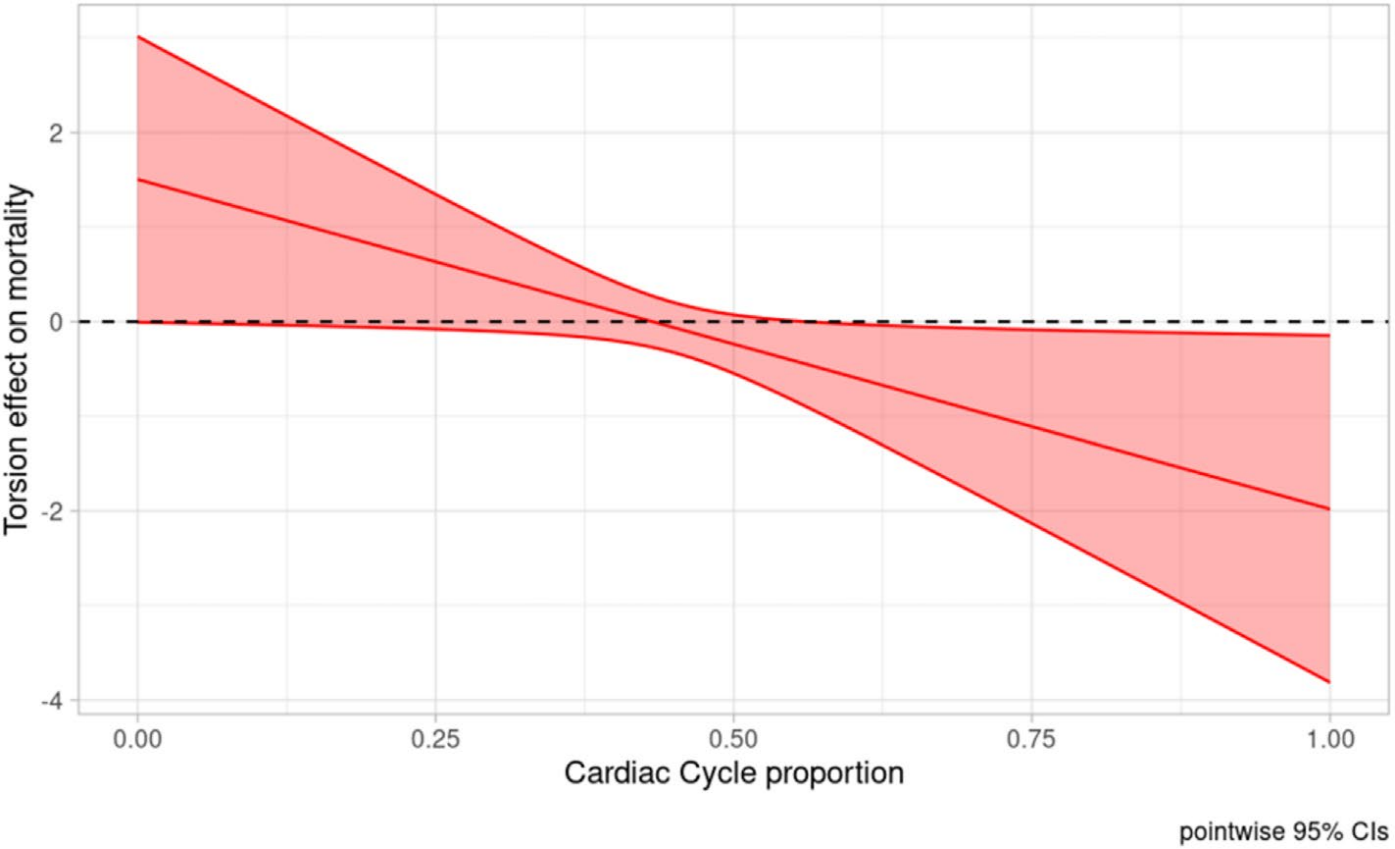
Left ventricular torsion effect on mortality with its confidence interval. The horizontal dashed line corresponds to 0. Effect is significant at the cardiac cycle proportion where the confidence interval does not contain 0. The y‐axis shows the log‐transformed odds ratio [log(OR)] of mortality.

The analysis showed that stronger diastolic untwist was significantly associated with lower mortality (Figure 4, global *p*‐value: 0.1). Despite the global p being greater than 0.05, it is common for our functional coefficient β(t) to have statistically significant regions without the entire function being globally significant (in our case, only the untwist part being significant).

**FIGURE 5:**
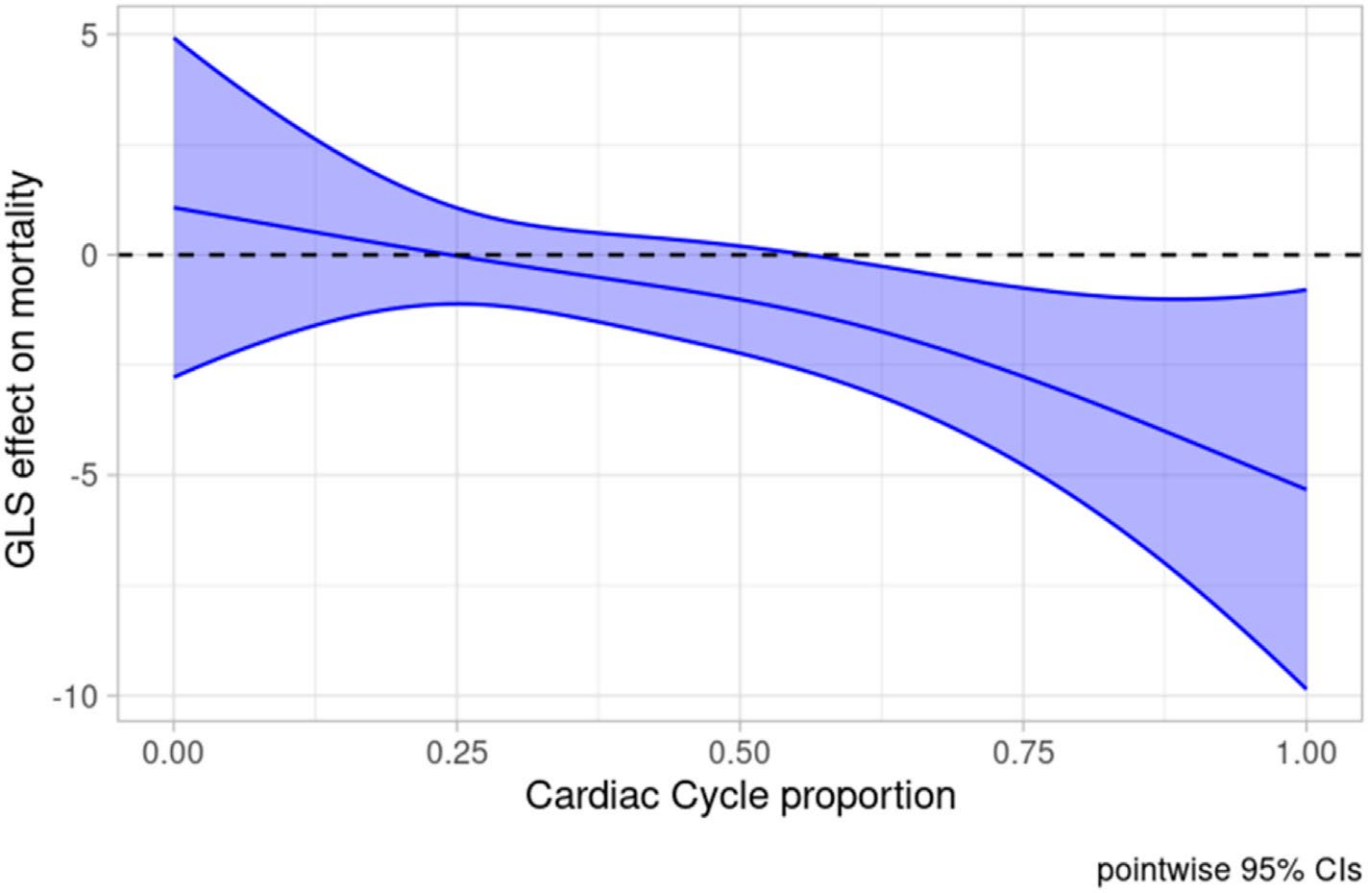
Global longitudinal strain effect on mortality with its confidence interval. The horizontal dashed line corresponds to 0. Effect is significant at the cardiac cycle proportion where the confidence interval does not contain 0. The y‐axis shows the log‐transformed odds ratio [log(OR)] of mortality. GLS, global longitudinal strain.

On the other hand, more negative GLS during diastole was associated with increased mortality (Figure 5, global *p*‐value 0.03).

A sensitivity analysis adjusting for age was also performed. This worsened overall model fit (higher AIC: 38.1 vs. 36.2) and had minimal impact on explained deviance. Therefore, the unadjusted model was retained as the primary analysis, with results from the age‐adjusted model presented in the Supplementary Material.

Our analysis implies that patients with 5% more negative GLS values after *t* = 0.5 of the cardiac cycle are estimated to have 1.57 (95% Wald CI: 1.13–2.18) times higher odds for death in the first 25 months (age‐adjusted: 1.61 (1.06–2.44)) compared to patients with the average GLS strain curve (Figure S4).

For LV torsion, a 5% degree increase (more positive) after t = 0.5 relative to the mean LV torsion curve implies lower odds for 25 months mortality by a factor of 0.85 (0.73–0.99; age‐adjusted: 0.84; 0.71–1.00) (Figure S5).

## 4 DISCUSSION

Our main findings revealed that higher untwist values were protective against a decrease in SBP and linked to better prognosis. Conversely, more negative diastolic GLS values were associated with increased mortality.

Untwist values measure the difference in rotation between the apex and the base of the left ventricle. Higher untwist values indicate diastolic clockwise rotation of the apex surpasses the counterclockwise rotation of the LV base. Maintaining a high untwist creates a diastolic depression in the LV, generating a suction effect that facilitates blood flow from the left atrium to the LV during diastole. On the contrary, low untwist values result in poorer LV filling, as the suction effect drawing blood from the left atrium is diminished.

During hemodialysis, intravascular hypovolemia occurs because the rate of fluid withdrawal exceeds the rate of refilling from the interstitium into the vascular space. The consequent reduced venous return leads to inadequate filling of the LV, resulting in a drop of cardiac output (Sars et al., 2020) and SBP. Interestingly, our study found that untwist, rather than longitudinal relaxation, was a determinant of SBP. It has long been hypothesized that the untwist of myocardial helices was the driver of diastolic suction and is the main compensatory mechanism for maintaining stroke volume during hypovolemia (Shibata et al., 2021). Furthermore, the untwist effect was shown to be more prominent during isovolumic relaxation, aligning with our findings (Hodt et al., 2015). Hemodialysis patients often experience significant LV hypertrophy, which tends to preserve ejection fraction but alters diastolic function (Smiseth et al., 2025; Assa et al., 2013; Foley et al., 2010; McCullough et al., 2016). Consequently, these patients exhibit baseline filling impairments. With the added effect of induced hypovolemia, hemodialysis patients naturally become exposed to a high risk of hypotension. Surprisingly, untwist usually remains normal in patients with diastolic dysfunction and normal EF, irrespective of the presence or absence of heart failure (Nagueh, 2020; Wang et al., 2007).

This suggests that untwist is the last cardiac compensatory mechanism allowing the maintenance of adequate SBP in the face of hypovolemia when other cardiac mechanisms are compromised in this subset of patients.

### 4.1 Prognosis

The all‐cause mortality rate was low (8%) in our patients compared to previous studies that examined similar patients with normal LVEF (Liu et al., 2013; Zhang et al., 2024). Interestingly, only diastolic strain value parameters were associated with prognosis despite including strain value from the whole cardiac cycle. For instance, patients with better untwist and those with better diastolic longitudinal relaxation experienced better outcomes in our study. Many studies have shown that echocardiographic parameters related to the severity of diastolic dysfunction were associated with worse outcomes (Nakagawa et al., 2022; Wang et al., 2024). However, these studies used only single‐point parameters, unlike our study, which included the entire strain curves as predictors. In fact, several other studies have suggested the superiority of this method (Bouchahda et al., 2025; Gruca et al., 2024; Remme et al., 2024), as the strain waveform contains more information than just one value at a given time.

Our findings may improve risk stratification and more tailored therapeutic strategies for hemodialysis patients with mildly reduced or preserved LVEF. Integrating full‐curve analyses of untwist and global longitudinal strain into routine echocardiographic assessment offers a novel, more sensitive approach to managing intradialytic hypotension and optimizing outcomes in this population, particularly where conventional measures may be insufficient.

Future research should prioritize the development and evaluation of interventions aimed at enhancing diastolic untwisting, which holds significant potential to improve hemodynamic stability and clinical outcomes during dialysis in these vulnerable patients.

Study Limitations: This study has several limitations. First, the small number of events made it challenging to adjust mortality to other classical patient characteristics other than LV mechanics. Second, hypotension events were not explored, as only three consecutive hemodialysis sessions were recorded. Although incorporating myocardial work analysis would have added value, particularly by integrating blood pressure with global longitudinal strain, this was not feasible due to limitations in our echocardiographic software. Longitudinal strain rate data were also not collected, which may have further enriched the analysis of diastolic function. Future studies should consider including these parameters to offer a more comprehensive evaluation of myocardial mechanics in this population.

## 5 CONCLUSION

Untwist plays a crucial role in maintaining SBP during hemodialysis. Furthermore, better untwist and better diastolic longitudinal relaxation were associated with better outcome.

## AUTHOR CONTRIBUTIONS

NB, WR, MYK, and AN contributed to data collection and patient management. FS performed the statistical analysis. NB, KH, NBM, MBS, and SA contributed to the interpretation of results. MHI, SHa, HM, and MBM contributed to the literature review and manuscript drafting. HS and NB provided critical revision and final approval of the manuscript. All authors read and approved the final version of the manuscript.

## FUNDING INFORMATION

None to declare.

## DISCLOSURES

None to report.

## Supporting information


Data S1:

## Data Availability

Stored in repository: Data and R code will be available at https://github.com/fabian‐s/LV‐untwist‐SBP.

## References

[phy270609-bib-0001] Assa, S. , Hummel, Y. M. , Voors, A. A. , Kuipers, J. , Groen, H. , De Jong, P. E. , Westerhuis, R. , & Franssen, C. F. M. (2013). Changes in left ventricular diastolic function during hemodialysis sessions. American Journal of Kidney Diseases, 62, 549–556. 10.1053/j.ajkd.2013.02.356 23548554

[phy270609-bib-0002] Badano, L. P. , & Muraru, D. (2019). Twist mechanics of the left ventricle. Circulation. Cardiovascular Imaging, 12, e009085. 10.1161/CIRCIMAGING.119.009085 31002264

[phy270609-bib-0003] Bouchahda, N. , Bader, M. , Najjar, A. , Mghaieth Zghal, F. , Sassi, G. , Mourali, M. S. , & Ben Messaoud, M. (2025). Effect of digoxin vs beta‐blockers on left atrial strain for heart rate‐controlled Atrial fibrillation: The DIGOBET‐AF randomized clinical trial. American Journal of Cardiovascular Drugs: Drugs, Devices, and Other Interventions, 25, 411–418. 39725795 10.1007/s40256-024-00705-w

[phy270609-bib-0004] Crainiceanu, C. M. , Goldsmith, J. , Leroux, A. , & Cui, E. (2024). Functional data analysis with R. Chapman and Hall/CRC.

[phy270609-bib-0005] Flythe, J. E. , Xue, H. , Lynch, K. E. , Curhan, G. C. , & Brunelli, S. M. (2015). Association of mortality risk with various definitions of intradialytic hypotension. Journal of the American Society of Nephrology, 26, 724–734. 10.1681/ASN.2014020222 25270068 PMC4341481

[phy270609-bib-0006] Foley, R. N. , Curtis, B. M. , Randell, E. W. , & Parfrey, P. S. (2010). Left ventricular hypertrophy in new hemodialysis patients without symptomatic cardiac disease. Clinical Journal of the American Society of Nephrology, 5, 805–813. 10.2215/CJN.07761109 20378644 PMC2863966

[phy270609-bib-0007] Goldsmith, J. , Bobb, J. , Crainiceanu, C. M. , Caffo, B. , & Reich, D. (2011). Penalized functional regression. Journal of Computational and Graphical Statistics, 20, 830–851. 10.1198/jcgs.2010.10007 22368438 PMC3285536

[phy270609-bib-0008] Goldsmith, J. , Scheipl, F. , Huang, L. , Wrobel, J. , Di, C. , Gellar, J. , Harezlak, J. , McLean, M. W. , Swihart, B. , Xiao, L. , Crainiceanu, C. , Reiss, P. T. , & Cui, E. (2010). Refund: Regression with functional data.

[phy270609-bib-0009] Gruca, M. M. , Slivnick, J. A. , Singh, A. , Cotella, J. I. , Subashchandran, V. , Prabhu, D. , Asch, F. M. , Siddiki, M. , Gupta, N. , Mor‐Avi, V. , Su, J. L. , & Lang, R. M. (2024). Noninvasive assessment of left ventricular end‐diastolic pressure using machine learning–derived phasic left atrial strain. European Heart Journal Cardiovascular Imaging, 25, 18–26. 10.1093/ehjci/jead231 37708373

[phy270609-bib-0010] Hodt, A. , Hisdal, J. , Stugaard, M. , Stranden, E. , Atar, D. , & Steine, K. (2015). Increased LV apical untwist during preload reduction in healthy humans: An echocardiographic speckle tracking study during lower body negative pressure. Physiological Reports, 3, 1–12. 10.14814/phy2.12330 PMC4393164 25802362

[phy270609-bib-0011] Liu, Y. W. , Su, C. T. , Sung, J. M. , Wang, S. P. H. , Su, Y. R. , Yang, C. S. , Tsai, L. M. , Chen, J. H. , & Tsai, W. C. (2013). Association of left ventricular longitudinal strain with mortality among stable hemodialysis patients with preserved left ventricular ejection fraction. Clinical Journal of the American Society of Nephrology, 8, 1564–1574. 10.2215/CJN.10671012 23704303 PMC3805079

[phy270609-bib-0012] McCullough, P. A. , Chan, C. T. , Weinhandl, E. D. , Burkart, J. M. , & Bakris, G. L. (2016). Intensive hemodialysis, left ventricular hypertrophy, and cardiovascular disease. American Journal of Kidney Diseases, 68, S5–S14. 10.1053/j.ajkd.2016.05.025 27772643

[phy270609-bib-0013] Nagueh, S. F. (2020). Left ventricular diastolic function: Understanding pathophysiology, diagnosis, and prognosis with echocardiography. JACC Cardiovasc Imaging, 13, 228–244. 30982669 10.1016/j.jcmg.2018.10.038

[phy270609-bib-0014] Nakagawa, A. , Yasumura, Y. , Yoshida, C. , Okumura, T. , Tateishi, J. , Yoshida, J. , Seo, M. , Yano, M. , Hayashi, T. , Nakagawa, Y. , Tamaki, S. , Yamada, T. , Kurakami, H. , Sotomi, Y. , Nakatani, D. , Hikoso, S. , Sakata, Y. , Sakata, Y. , Hikoso, S. , … Hikoso, S. (2022). Predictors and outcomes of heart failure with preserved ejection fraction in patients with a left ventricular ejection fraction above or below 60%. Journal of the American Heart Association, 11, e025300. 10.1161/JAHA.122.025300 35904209 PMC9375469

[phy270609-bib-0015] Remme, E. W. , Inoue, K. , & Smiseth, O. A. (2024). Machine learning in diastolic dysfunction: Left atrial strain trace superior to single points for estimation of filling pressure. European Heart Journal. Cardiovascular Imaging, 25, 27–28. 10.1093/ehjci/jead257 PMC10735308 37818845

[phy270609-bib-0016] Sabatino, J. , Castaldi, B. , & Di Salvo, G. (2021). How to measure left ventricular twist by two‐dimensional speckle‐tracking analysis. European Heart Journal Cardiovascular Imaging, 22, 961–963. 10.1093/ehjci/jeab108 34160033

[phy270609-bib-0017] Sars, B. , Van Der Sande, F. M. , & Kooman, J. P. (2020). Intradialytic hypotension: Mechanisms and outcome. Blood Purification, 49, 158–167. 10.1159/000503776 31851975 PMC7114908

[phy270609-bib-0018] Scheipl, F. , Staicu, A.‐M. , & Greven, S. (2015). Functional additive mixed models. Journal of Computational and Graphical Statistics, 24, 477–501. 10.1080/10618600.2014.901914 26347592 PMC4560367

[phy270609-bib-0019] Shibata, S. , Hirabuki, K. , Hata, N. , Suzuki, R. , Suda, T. , Uechi, T. , & Hirasawa, A. (2021). Pivotal role of heart for orthostasis: Left ventricular untwisting mechanics and physical fitness. Exercise and Sport Sciences Reviews, 49, 88–98. 10.1249/JES.0000000000000247 33720910

[phy270609-bib-0020] Smiseth, O. A. , Rider, O. , Cvijic, M. , Valkovič, L. , Remme, E. W. , & Voigt, J.‐U. (2025). Myocardial strain imaging: Theory, current practice, and the future. JACC. Cardiovascular Imaging, 18, 340–381. 39269417 10.1016/j.jcmg.2024.07.011

[phy270609-bib-0021] Wang, J. , Khoury, D. S. , Yue, Y. , Torre‐Amione, G. , & Nagueh, S. F. (2007). Left ventricular untwisting rate by speckle tracking echocardiography. Circulation, 116, 2580–2586. 10.1161/CIRCULATIONAHA.107.706770 17998458

[phy270609-bib-0022] Wang, N. , Rueter, P. , Ng, M. , Chandramohan, S. , Hibbert, T. , O'Sullivan, J. F. , Kaye, D. , & Lal, S. (2024). Echocardiographic predictors of cardiovascular outcome in heart failure with preserved ejection fraction. European Journal of Heart Failure, 26, 1778–1787. 10.1002/ejhf.3271 38714362

[phy270609-bib-0023] Wood, S. N. (2017). Generalized additive models. Chapman and Hall/CRC.

[phy270609-bib-0024] Zhang, Y. , Guo, X. , Chen, S. , Wang, Y. , Li, J. , Sun, X. , & Huang, X. (2024). Left ventricular geometry characteristics and clinical outcomes in hemodialysis patients with heart failure with preserved ejection fraction. BMC Cardiovascular Disorders, 24, 327. 10.1186/s12872-024-03985-x 38926680 PMC11210017

