# Supplementary material for: Left ventricular untwist determines intradialytic hemodynamics and outcomes in mildly reduced and preserved ejection fraction patients
Source: Physiol Rep. 2025 Nov 2;13(21):e70609. doi: 10.14814/phy2.70609 (PMC12580422; doi:10.14814/phy2.70609)
Supplement: Supplementary file 1 [file PHY2-13-e70609-s001.odt]

Figure 1 : A representative example of global longitudinal strain acquisition from a patient.

Figure 2: A representative example of left ventricular torsion acquisition from a patient.

Scalar-on-function logistic regression models predicting all-cause mortality

| Model | AIC | Deviance Explained (%) |
| --- | --- | --- |
| GLS + LV Torsion (unadjusted) | 36.2 | 5.78 |
| GLS + LV Torsion + Age (adjusted) | 38.1 | 7.22 |

- Deviance explained represents the proportion of deviance accounted for by the model.

- A lower AIC indicates better model fit.

- Adding age as a covariate increased deviance explained slightly but worsened the overall model fit as indicated by the higher AIC.

Figure 3 : Left panel: Global longitudinal strain effect on mortality with its confidence interval after adjustment for age. Right panel: left ventricular torsion effect on mortality with its confidence interval after adjustment for age. The horizontal dashed line corresponds to 0. Effect is significant at the cardiac cycle proportion where the confidence interval does not contain 0. Partial effect shown on log-odds scale. GLS: global longitudinal strain.

Figure 4: Example of a patient with mean GLS (black solid curve) and a patient with a 5% relative decrease in GLS after t=0.5 (red dashed curve).

Figure 5: Example of a patient with mean LV torsion  (black solid curve) and a patient with a 5% relative increase in LV untwist after t=0.5 (red dashed curve).
